# Optimizing Autonomous Taxi Deployment for Safety at Skewed Intersections: A Simulation Study

**DOI:** 10.3390/s25113544

**Published:** 2025-06-04

**Authors:** Zi Yang, Yaojie Yao, Liyan Zhang

**Affiliations:** 1 School of Automation, Nanjing University of Science and Technology, Nanjing 210044, China; 2 School of Intelligent Manufacturing, Nanjing University of Science and Technology Zijin College, Nanjing 210023, China

**Keywords:** autonomous taxi, intersection safety, skewed intersection

## Abstract

This study optimizes the deployment of autonomous taxis for safety at skewed intersections through a simulation-based approach, identifying an optimal penetration rate and control strategies. Here, we investigate the safety impacts of autonomous taxis (ATs) at such intersections using a simulation-based approach, leveraging the VISSIM traffic simulation tool and the Surrogate Safety Assessment Model (SSAM). Our study identifies an optimal AT penetration rate of approximately 0.5–0.7, as exceeding this range may lead to a decline in safety metrics such as TTC and PET. We find that connectivity among ATs does not linearly correlate with safety improvements, suggesting a nuanced approach to AT deployment is necessary. The “Normal” control strategy, which mimics human driving, shows a direct proportionality between AT penetration and TTC, indicating that not all levels of automation enhance safety. Our conflict analysis reveals distinct patterns for crossing, lane-change, and rear-end conflicts under various control strategies, underscoring the need for tailored approaches at skewed intersections. This research contributes to the discourse on AT safety and informs the development of traffic management strategies and policy frameworks that prioritize safety and efficiency in the context of skewed intersections.

## 1. Introduction

The emergence of autonomous taxis (ATs) represents a pivotal development in the advancement of urban shared mobility. As cities grapple with congestion and safety concerns, the potential of ATs to alleviate these issues is increasingly recognized. ATs are poised to revolutionize urban mobility, offering scalable solutions to urban transportation challenges. Their deployment is driven by economic incentives, such as cost reductions and operational efficiencies, and facilitated by controlled urban environments that are conducive to autonomous vehicle (AVs) technology.

The emergence of ATs represents a pivotal development in the advancement of urban shared mobility. As cities grapple with congestion and safety concerns, the potential of ATs to alleviate these issues is increasingly recognized. Several factors position ATs to precede privately owned AVs in large-scale deployment. The service-oriented model of ATs enables iterative improvements through centralized fleet management, such as coordinated routing and platooning, contrasting with the fragmented adoption required for private AVs. Economically, AT deployments are driven by cost reductions and operational efficiencies, incentivizing strategies that minimize downtime through rapid passenger turnover and aggressive scheduling. Regulatory frameworks for ATs can also be tailored to their operational nuances, unlike the broader policies needed for private AVs.

However, these very advantages introduce unique challenges at skewed intersections. As shared mobility platforms, ATs exhibit higher traffic density due to frequent passenger pick-up/drop-off activities and centralized control, resulting in irregular lane-changing patterns, abrupt decelerations, and competitive merging behaviors. Such dynamics are exacerbated in skewed geometries with uneven traffic distribution, where asymmetric flows amplify conflicts during turning maneuvers. Furthermore, economic pressures to maximize fleet utilization often prioritize efficiency over conservative safety margins, a trade-off less prevalent in private AVs designed for individualized user preferences. The interplay of centralized coordination, frequent stops, and geometric irregularities necessitates a specialized evaluation of AT safety impacts, as general AV studies frequently overlook these fleet-specific operational complexities.

As AT and AV services move from controlled testing environments into real-world applications, a pivotal question arises: How do these technologies impact the safety of urban transportation systems? This question is of paramount importance to users, legislators, and operators alike. Despite extensive research on AV safety, the specific dynamics of skewed intersections, where traffic flow is unevenly distributed, have been largely overlooked. These intersections present unique challenges that necessitate a specialized evaluation of AV performance. Unlike standard intersections, skewed intersections have asymmetrical lane configurations, which result in uneven distribution of traffic flow in different directions. Due to the lack of sight distance at skewed intersections, vehicles need more time to cross the intersection. This leads to increased exposure time to conflicting traffic, which increases the risk of accidents.

Extensive research has been conducted on the development and refinement of control strategies for autonomous and mixed-traffic intersections. Notably, Namazi et al. (2019) [1] conducted a systematic review of intelligent intersection management systems, categorizing approaches into rule-based, optimization, hybrid, and machine-learning paradigms. Khayatian et al. (2020) [2] extended this discourse with an exhaustive survey on intersection management for Connected Autonomous Vehicles (CAVs), covering interfaces, algorithms, and communication protocols. Research has also explored the safety and efficiency implications of AVs at intersections, with Zhang et al. (2020) [3] presenting an autonomous intersection control approach for connected AVs, integrating global optimization scheduling to mitigate collision risks. Chen et al. (2020) [4] introduced an autonomous intersection management algorithm that incorporates pedestrian dynamics to maximize throughput. The role of Vehicle-to-Everything (V2X) communication in enhancing intersection safety has been a notable trend in recent research, with Dresner and Stone (2008) [5] proposing a multiagent system approach for coordinating AVs through intersections, relying on detailed communication protocols. Aoki and Rajkumar (2022) [6] introduced a Distributed Synchronous Intersection Protocol (DSIP) and a Cooperative Perception-based High-Definition Map (CP-HD Map) to manage mixed traffic environments. Li et al. [7] proposed an energy management strategy (EMS) for a fuel cell hybrid electric bus (FCHEB) based on a deep deterministic policy gradient (DDPG) algorithm. The information exchange between the vehicle system and the network layer is realized through CPS to obtain the environmental conditions of vehicle operation, which is the first time that future terrain information is introduced into the framework. The proposed strategy realizes an effective integration with the information–physical system and achieves a trade-off between hydrogen consumption and energy durability.

However, while the literature has extensively covered symmetric traffic scenarios, the dynamics of skewed intersections have been largely overlooked. Skewed intersections, characterized by uneven traffic distribution, present unique challenges that require specialized evaluation and management strategies. Most studies assume a fully autonomous traffic environment, which may not reflect the gradual penetration of AVs and the mixed traffic conditions prevalent in real-world settings. The transition phase, where AVs coexist with conventional vehicles, is critical and requires further investigation. Traditional safety metrics such as Time-to-Collision (TTC) and Post-Encroachment Time (PET) are widely used, but there is a need to incorporate more advanced metrics to provide a comprehensive assessment of safety in mixed traffic environments. Additionally, there is a lack of detailed operational guidelines and implementation steps for deploying ATs in real-world scenarios. Practical insights into how to optimize penetration rates and connectivity levels based on specific intersection characteristics are needed.

This study addresses these gaps by focusing on the safety implications of ATs at skewed intersections. Using a simulation-based approach with VISSIM and the Surrogate Safety Assessment Model (SSAM), we provide a nuanced analysis of the interaction between AT control logic and traffic dynamics under varied penetration rates and connectivity scenarios. Our findings contribute to the discourse on AT safety and inform the development of traffic management strategies and policy frameworks that prioritize safety and efficiency in the context of skewed intersections.

The paper is structured as follows: After this introduction, we review the related work in the field. Subsequently, we detail our simulation settings for both symmetric and skewed intersections. The results section presents our findings on the impact of automation and connectivity on safety at these intersections, followed by a discussion on the implications for urban planning and transportation policy. We conclude with recommendations for future research and the development of shared mobility systems that prioritize safety and efficiency, with a focus on skewed intersections.

## 2. Related Work

### 2.1. Intersection Management Systems

The domain of autonomous and mixed-traffic intersection management has been extensively examined within the academic literature, with a pronounced emphasis on the development and refinement of control strategies for such critical transportation nodes. Notably, Namazi et al. (2019) [1] executed a meticulous systematic review that centered on the evolution of intelligent intersection management systems within the autonomous vehicle sphere. Their analysis encompassed a comprehensive corpus of 105 seminal studies spanning the period from 2008 to 2019, methodically categorized by their underlying approaches: rule-based, optimization, hybrid, and machine-learning paradigms. This is the first time that heterogeneous traffic flows (a mixture of human driving and autonomous driving) are included in a system evaluation framework, breaking through the limitation of traditional studies that focus only on a single vehicle type. Subsequently, Khayatian et al. (2020) [2] extended the discourse by conducting an exhaustive survey on intersection management tailored for CAVs. Their scrutiny was multifaceted, delving into intersection management interfaces, scheduling algorithms, wireless communication protocols, vehicle modeling, conflict resolution strategies, and the orchestration of multiple intersections in concert. In focusing on the practicality of complex urban road scenarios, Li et al. (2021) [8] further contributed to the discourse by offering a synthesized overview of the planning and decision-making technologies that are pivotal at intersections, with a particular emphasis on the nuanced dynamics of CAVs. Their exposition included a spectrum of methodologies, ranging from graph-theoretic models to predictive analytics, optimization heuristics, and machine-learning frameworks. The framework combining hierarchical classification provides researchers with a panoramic view of CAV behavioral decision-making in intersection scenarios. However, this approach is highly dependent on data quality and labeling. The model relies on high-quality GPS trajectory data and requires fine meshing and topological relationship labeling. In scenarios with sparse data or incomplete labeling, such as rural areas, the performance may be significantly degraded. In a parallel vein, Zhong, Nejad, and Lee (2021) [9] presented a sweeping survey of the state-of-the-art in autonomous intersection management (AIM), dissecting the multi-tiered design considerations that span from corridor-level coordination to intersection-specific management and granular vehicle control mechanisms. Their review was particularly attuned to the intricacies of conflict detection, the establishment of priority rules, and the computational challenges inherent in AIM systems. It integrates autonomous (AM) and semi-autonomous (SAM) intersection management systems into a unified classification framework and proposes a three-dimensional classification model based on “decision level–control granularity–communication range”, which builds clear cognitive coordinates for domain research. A progressive deployment route is proposed for mixed traffic flow (coexistence of human drivers and CAVs). However, the depth of its validation is limited, and the actual road test projects of Waymo, Baidu, and other companies are only described in technical principles, which weakens the value of engineering guidance. At the same time, the theoretical model is oversimplified, adopting a linear game model to predict human driving behavior, ignoring the nonlinear characteristics of rapid acceleration and deceleration in the IDM follow-up model. Building upon this foundation, Al-Turki, Ratrout, Rahman, and Assi (2022) [10] offered a pointed critical review that focused on the prospective control methodologies for signalized intersections within mixed traffic environments that include both AVs and conventional vehicles (RVs). It incorporates reinforcement learning algorithms (e.g., DQN) and classical traffic flow theories, such as Webster’s signal timing model, into a unified analysis system, which provides researchers with a panoramic view of technology evolution. However, it also has the disadvantage of verifying the theoretical model only through MATLAB/SUMO simulation and lacks the support of real road test data. Concurrently, Gholamhosseinian and Seitz (2022) [11] conducted an extensive survey on the cooperative management of intersections for a diverse array of connected vehicles, factoring in the operational nuances of road and rail vehicles, as well as the safety considerations for vulnerable road users, such as cyclists, scooter riders, and pedestrians. It proposes a Nash–Stackelberg hybrid game model to solve the priority conflict between CAV and human-driven vehicle (HV), and reduces the communication delay from 120 ms to 35 ms in the centralized system; its main shortcomings are the insufficient depth of the mixed traffic scenarios, the adoption of a simplified IDM follow-up model to model the HV behaviors, and the failure to take into account the driving styles (aggressive/conservative) on the game strategy influence on the game strategy, resulting in the deviation of the simulation results from the actual road test.

### 2.2. Autonomous Intersection Control

In the realm of intersection management, a simplifying assumption often employed is the consideration of fully autonomous traffic, thereby rendering all vehicles controllable and responsive to a centralized control system. This approach has been predominantly utilized to enhance operational efficiency, with numerous studies concentrating on the cooperative control mechanisms that can be implemented at intersections to achieve optimal traffic flow. For instance, Zhang et al. (2020) [3] presented a robust autonomous intersection control (AIC) approach tailored for connected autonomous vehicles. This method integrates global optimization scheduling to mitigate the risk of collisions under variable communication conditions, thereby ensuring traffic efficiency. Chen et al. (2020) [4] introduced AIM-ped, an autonomous intersection management algorithm that incorporates the dynamics of both vehicles and pedestrians, aiming to maximize throughput through the synergistic application of max pressure control. It combines the Multi-Intelligent Deep Deterministic Policy Gradient (MADDPG) algorithm with the prioritized experience playback mechanism, and proposes the PER-MADDPG framework, which effectively solves the convergence speed problem in sparse reward scenarios. And it constructs a hybrid traffic flow model that includes the heterogeneity of human driving behaviors, integrating the IDM follow-me model with the driving characteristic parameters of the NGSIM dataset. The main shortcoming of this approach is that the proposed PER-MADDPG algorithm does not evaluate the marginal cost relationship between the deployment density of roadside units (RSUs) and the improvement of access efficiency, and it lacks a cost–benefit indicator similar to the quantization of box-and-line diagrams. Lu et al. (2022) [12] conducted a comparative computational study on the efficiency of futuristic intersections designed exclusively for connected, autonomous, and centrally managed vehicles. The study evaluated three distinct control strategies, each aimed at minimizing overall system delay by optimizing the trajectory of individual vehicles. The research combines the cellular automata model with deep-reinforcement learning (DRL), proposes a two-layer framework of “hybrid traffic flow simulation-multi-intelligence collaborative decision-making”, and breakthroughs of the integration of traffic engineering theories (Webster’s delay model) and computer science methods (PPO reinforcement-learning algorithms), to establish a vehicle trajectory conflict prediction model, which greatly enhances the computational efficiency. However, it adopts a simplified IDM model for simulating human driving behavior, ignoring the heterogeneity of driving styles, which may lead to an increase in conflict prediction error. Wang, Cai, and Lu (2020) [13] proposed a cooperative autonomous traffic organization method for connected automated vehicles within multi-intersection road networks, addressing the complexities of spatiotemporal constraints, multiple trajectory optimization objectives, and diverse decision-making behaviors in dynamic traffic scenarios. It proposes a two-layer collaborative framework of “centralized scheduling-distributed gaming”, realizes global traffic balancing at multiple intersections through V2X communication, develops the Spatiotemporal Corridor model, optimizes the vehicle trajectory planning and signal timing jointly, and solves the problem of weight allocation in multi-objective optimization by introducing the virtual queue length as a feedback variable.

Similarly, by introducing the “virtual buffer zone” mechanism, Wang, Wen, and Chao (2021) [14] unveiled Roadrunner+, an Autonomous Intersection Management (AIM) system that collaborates with CAVs and pedestrians, considering the impact of spillback from downstream intersections. This system is engineered to manage collision avoidance, facilitate efficient CAV control, and tackle challenges posed by pedestrian crossings and congestion-induced blockages. It proposes a “CAV–Pedestrian–Overflow” ternary cooperative control architecture, which dynamically couples the pedestrian trajectory prediction model (based on LSTM-GAN) with vehicle trajectory planning (MPC algorithm) for the first time. The framework realizes a 42% reduction in average pedestrian waiting time in the SUMO simulation. Wu et al. (2022) [15] explored the optimization of entrance and exit lanes within autonomous intersection management for connected and automated vehicles. They proposed the concept of “all-direction” lanes to accommodate left-turn, through, and right-turn traffic, and developed optimization techniques for entering times and route selections. Vitale, Kolios, and Ellinas (2022) [16] introduced an innovative framework for managing intersections in the context of CAVs, accounting for uncertainties in vehicle positioning. It proposes a probabilistic occupancy grid-based vehicle localization uncertainty quantification framework, combining Bayesian filtering and conflict probability prediction algorithms, which maintains 91.3% conflict detection accuracy when the standard deviation of the localization error is >1.5 m. The proposed framework is based on the “global path planning–local trajectory correction” dual-layer control architecture.

### 2.3. Autonomous Vehicle Decision-Making at Intersections

Several studies have specifically focused on the planning and decision-making processes of autonomous vehicles at intersections. Noh (2019) [17] proposed a decision-making framework for autonomous vehicles at road intersections, which is designed to address the challenges of navigating safely and efficiently amidst vehicles that may not adhere to traffic regulations. It proposes a multimodal decision-making framework, such as a synergistic mechanism, for, e.g., collision avoidance and offending vehicle detection. By introducing a dynamic risk assessment model, it effectively solves the problem of balancing safety and efficiency in traditional methods. This framework is significant for enhancing the safety of autonomous driving in complex traffic scenarios. Karthikeyan, Chen, and Hsiung (2022) [18] developed a deep reinforcement learning-inspired autonomous intersection management (DRLAIM) system. This system is innovative in its approach to improving the efficiency and safety of traffic environments by using deep reinforcement learning to make dynamic decisions that adapt to the flow of traffic.

Xia, Xing, and He (2022) [19] presented a planning framework based on the partially observable Markov decision process (POMDP) for autonomous vehicles at intersections. This framework is particularly relevant for situations where traffic signs are not present, as it ensures social compliance and optimizes the motion responses of autonomous vehicles to fit within the expectations of human drivers and traffic norms. It proposes an interaction planning mechanism that introduces a new decision logic in the dynamic coordination of unsignalized intersections, which improves the real-time and adaptive decision-making by dynamically predicting the intentions of other traffic participants. It also considers the interaction between manual and automatic driving, thus reducing conflicts caused by uncertainty in driver behavior. This adaptive design is relevant in the context of the gradually increasing proportion of mixed driving.

### 2.4. Vehicle-to-Everything (V2X) Communication in Intersection Management

The recognition of the pivotal role played by Vehicle-to-Everything (V2X) communication in the control of intersections has been a notable trend in recent research. Dresner and Stone (2008) [5] proposed a multi-agent system approach to coordinate the movement of autonomous vehicles through intersections, suggesting a reservation-based mechanism that relies on detailed communication protocols between drivers and intersections. This system is foundational in promoting a smooth and safe transition of traffic through intersections. Aoki and Rajkumar (2022) [6] introduced a Distributed Synchronous Intersection Protocol (DSIP) and a Cooperative Perception-based High-Definition Map (CP-HD Map) to manage intersections in mixed traffic environments. Their approach utilizes dynamic decision-making mechanisms and Vehicle-to-Vehicle (V2V) communications to adapt vehicle behaviors and share detected object information, which is essential for reducing collision risks and enhancing intersection safety. Furthermore, the concept of a “Cyber Traffic Light” was put forth by Aoki and Rajkumar (2022) [20], which is a vehicular communication-based traffic management system for autonomous vehicles at dynamic intersections. This system is designed to allocate green periods for vehicles coming from multiple directions, ensuring safe cooperation and collaboration among autonomous driving vehicles, thereby contributing to the overall safety and efficiency of intersection traffic management. It significantly improves the global situational awareness of mixed traffic flow by sharing real-time intersection dynamic information. And for the realistic conditions of coexistence of human-driven and self-driven vehicles, it proposes hierarchical decision-making mechanisms, such as priority dynamic allocation and safety gap calculation, to solve the problem that traditional signal control is unable to adapt to the differences in mixed-driving behaviors. Its advantage lies in the possibility of achieving a more realistic interaction strategy, which is superior to purely theoretical optimization algorithms. However, it does not sufficiently discuss fault tolerance mechanisms under communication interruption or sensor failure. Jia [21] proposed a health-aware EMS framework for FCHEB based on a two-delay deep deterministic policy gradient (TD3) algorithm. The environmental and road ahead information acquired through vehicle sensors, GPS, and GIS is integrated into the state-of-the-art TD3 algorithm, and optimal power distribution and overall vehicle economy of FCHEB are effectively improved. Further, Jia [22] has proposed an EMS that integrates future road information and cabin comfort control to achieve optimal energy distribution between different on-board energy systems. The impact of multi-source information fusion and ACS control on EMS is quantified, and FCB energy efficiency is improved by on-board sensors and vehicle cloud infrastructure, thus further reducing overall vehicle operating costs.

### 2.5. Safety Implications of Autonomous Vehicles at Intersections

However, a critical issue with the existing body of research is the underlying assumption of a fully autonomous traffic environment, which often overlooks the transitional phase where AVs coexist with conventional vehicles. This assumption may not accurately reflect the complexities of real-world traffic, where a mix of vehicle types necessitates a more nuanced approach to intersection management. The primary focus of many studies on enhancing traffic efficiency, while valuable, may not sufficiently address the paramount concern of safety, which is essential for the successful integration and acceptance of AVs on the road. Mousavi et al. (2020) [23] shed light on this gap by investigating the potential of AVs to reduce accidents and improve overall traffic safety in scenarios that are currently dependent on human decision-making and adherence to traffic rules. Their findings underscore the importance of AVs in enhancing road safety, highlighting the need for further research in this area. It proposes multi-dimensional safety evaluation indexes, such as conflict time and trajectory deviation, combines traditional traffic safety theories with autonomous driving characteristics, and adopts a combination of real traffic datasets and microsimulation to establish a quantitative model for safety enhancement at signal-less intersections. The relationship between the penetration rate of automatic driving and the accident rate is verified by mixed traffic flow simulation. Pourjafari, Ghafari, and Ghaffari (2024) [24] directly addressed the critical issue of collision avoidance at unsignalized intersections for AVs through an online speed planning approach. They developed an interaction-aware prediction algorithm that leverages Long Short-Term Memory networks (LSTM) and Graph Neural Networks (GNN) to predict vehicle sequences at collision points and estimate safe traversal times. This innovative approach provides a significant advancement in the field by offering a proactive method to prevent collisions and ensure the safe navigation of AVs through intersections. However, it does not adequately discuss sensor noise, such as camera low-light failures or communication delays. If the algorithm relies on high-precision environmental sensing, its practical deployment may be limited by adverse weather conditions. Moreover, it only validates simple unsignalized intersections and does not address multi-lane skewed intersections or peak-hour mixed-flow scenarios. Virdi et al. (2019) [25] conducted a study to assess the safety improvements achieved by incrementally transitioning the vehicle fleet to CAVs. They used a calibrated microsimulation environment and a custom-developed algorithm for CAV behavior, introducing CAVs in increments and assessing safety performance using the SSAM. It realizes the quantitative assessment of traffic conflicts, such as tailgating and lane changing conflicts, by constructing a multi-vehicle interaction model through the VISSIM platform. The method breaks through the limitation of traditional accident statistics, relying on historical data. Its conflict reduction rate prediction provides a direct basis for policy formulation. And the simulation scenarios, such as different CAV penetration gradients and intersection types, are designed through the hierarchical control variable method, and dual indicators of TTC and PET are introduced to enhance the reliability of the assessment results.

A recent work by Xu, Wang (2025) [26] proposed a co-simulation-based safety assessment framework for CAV operations in large-scale test environments. The framework consists of three key components, the first being a high-fidelity test environment with varying road geometries and dynamic conditions, including weather variations and realistic traffic flows; the second being intelligent CAV functionality developed through deep reinforcement learning, and the third being a complex safety measure that utilizes integrated multi-type Bayesian hierarchical models to comprehensively assess risk factors and accident probabilities. Noting that safety benefits do not consistently correlate linearly with increased CAV penetration, the study identifies optimal stabilization of self-driving vehicles and human-driven vehicles at 70% CAV penetration and finds that roundabouts and signalized intersections account for more than 70% of conflicts involving CAVs. This work advances CAV safety validation by providing a more realistic, large-scale test environment that compensates for real-world testing limitations and allows for comprehensive safety evaluations in different scenarios, providing deeper insights into strategies for implementing CAVs in the near future.

In summary, the body of literature on autonomous vehicle intersection management has made significant strides, particularly in optimizing traffic flow and enhancing operational efficiency through various control strategies and decision-making frameworks. However, the majority of these studies have focused on symmetric traffic scenarios and have often assumed full autonomy, which may not reflect the gradual penetration of autonomous vehicles and mixed traffic conditions prevalent in real-world settings (as noted by Mousavi et al., 2020 [23]; Pourjafari et al., 2024 [24]). Moreover, the safety implications of autonomous vehicles at skewed intersections, which are characterized by asymmetric traffic patterns, have been notably understudied. This research gap is critical, as skewed intersections pose unique challenges that can influence the safety and efficiency of autonomous vehicle operations. Our study addresses this void by employing a simulation-based approach to assess the safety impacts of autonomous taxis at skewed intersections. By leveraging VISSIM for traffic simulation and the SSAM for safety evaluation, we provide a nuanced analysis of the interaction between autonomous vehicle control logic and traffic dynamics under varied penetration rates and connectivity scenarios. Our findings not only contribute to the understanding of autonomous vehicle safety in complex traffic environments but also inform the development of tailored traffic management strategies and regulatory frameworks for skewed intersections, thereby advancing the safe and efficient integration of autonomous vehicles into the urban transportation network.

## 3. Materials and Methods

### 3.1. Traffic Simulation

The traffic simulation is conducted in a Windows 10 operating system on a laptop with 16G RAM and Intel(R) i7-8565U CPU. The simulation software is VISSIM 4.3, a commonly used microsimulation tool in this domain. VISSIM is known for its ability to accurately replicate traffic circumstances with high reliability in real-world scenarios, including signalized intersections, which is crucial for the traffic simulation at intersections and the safety assessment afterwards. The models in VISSIM are calibrated and validated against real-world data, including the number of lanes, traffic volume, and car-following parameters, ensuring that the simulation results accurately reflect actual traffic conditions. Most importantly, VISSIM can be easily integrated with SSAM to derive indirect safety measures, which allows for a more comprehensive safety assessment by using surrogate safety measures that represent traffic stream safety. VISSIM’s Wiedemann car-following model can be accurately calibrated for mixed traffic (AV + HV) with parameters such as CC0 (standstill distance) and CC8 (acceleration). This granularity is essential to capture the subtle interactions at skewed intersections. VISSIM’s built-in signal logic editor supports complex phasing (e.g., 177-s cycles with phase-specific green times), which is critical for replicating real-world operations at the Tianyuan–Chuangpai crossing. SSAM’s TTC and PET algorithms have been empirically validated by FHWA studies to provide higher conflict recognition accuracy compared to rule-based alternatives. SUMO’s Krauss car-following model lacks the parameter depth of VISSIM’s Wiedemann model and is, therefore, less suitable for modeling nuanced audiovisual strategies (e.g., predictive coordination for All-Knowing). SUMO’s collision detection is binary (collision/no collision), whereas SSAM quantifies proximity collisions via TTC/PET, which is critical for prospective safety analysis. CARLA’s real-time 3D rendering and sensor simulations (e.g., LIDAR point clouds) require >10× the computational resources of VISSIM, making large-scale penetration rate scans (0.3–0.9) impractical. It takes 4.2 h to simulate 1 h of traffic flow in CARLA, compared to 18 min in VISSIM. CARLA prioritizes audio–visual perception training over macroscopic traffic dynamics and lacks a system-safety assessment tool like SSAM. To ensure the accuracy and reliability of our simulation, we followed a rigorous calibration and verification process. The specific steps and results are detailed below:

(1) Lane Configuration: We configured the VISSIM model to match the actual number of lanes and layout of the intersection, including dedicated left-turn and through lanes, and the alternating through-right lane. This is described in Section 3.2, Intersection Scenarios.

(2) Traffic Volume: We input the collected traffic volume data into the VISSIM model, ensuring that the simulated traffic flow matched the real-world data.

(3) Signal Timing: We set the signal timing in VISSIM to match the actual signal phases and durations. The actual signal cycle (177 s) and phase green hours (36 s for left turn, 62 s for straight ahead, and 98 s for right turn) at the intersection of Tianyuan Road and Zhuangpai Road were used directly.

(4) Car-Following Model: The car-following model uses Wiedemann 99 model as the basic model and uses different parameters to determine the driving characteristics of vehicles under different control strategies. There are four driving strategies, such as Rail-Safe, Cautions, Normal, and All-Knowing.

(5) Sensitivity Analysis: To further ensure the robustness of our model, we conducted a sensitivity analysis by varying the key parameters (penetration rate, connectivity, and control strategies). The results can be found in the Results and Discussion section.

By following these steps, we ensured that our VISSIM model was accurately calibrated and verified, providing a reliable basis for our simulation study. To model the driving behavior of robotaxis in our simulation, we selected the Wiedemann 99 car-following model, which is a widely used and well-validated model in traffic simulation studies. The Wiedemann 99 model is particularly suitable for our study due to its ability to capture a range of driving behaviors, from cautious to aggressive, through adjustable parameters. We will represent the driving behaviors of robotaxis using various parameter sets within the Wiedemann 99 model, a commonly utilized built-in car-following model in VISSIM. These different sets of parameters constitute different controlling strategies for autonomous vehicles, from the most cautious to the most radical. This flexibility allows us to simulate different control strategies for autonomous vehicles and to analyze their impact on safety performance at skewed intersections.

The Wiedemann 99 model parameters were chosen based on the CoEXist project, a European research initiative that has extensively studied the behavior of AVs in mixed-traffic environments. This project has developed computer simulations of road infrastructures in four distinct European cities—Helmond in the Netherlands, Stuttgart in Germany, Gothenburg in Sweden, and Milton Keynes in the United Kingdom. Specifically, the CoEXist project addresses the challenges of autonomous vehicles in urban environments. By assessing various car-following scenarios, speeds, and communication conditions, numerous plans were tested and compared. The CoEXist project defined four control strategies for AVs, ranging from the most conservative (“Rail Safe”) to the most aggressive (“All-knowing”) [27]. These strategies are based on real-world measurements and are designed to represent different levels of automation and driving behavior. Rail Safe is the most conservative, designed to mimic the behavior of a cautious driver who maintains a safe distance and follows traffic rules strictly. Cautious is moderately conservative, representing a driver who is careful but not overly cautious. Normal is designed to mimic the behavior of an average human driver, balancing safety and efficiency. All-Knowing is the most progressive, representing a highly advanced autonomous system that has perfect knowledge of the traffic environment and can make optimal decisions. The parameters for each strategy are detailed in Table 1. The meanings of the car-following parameters in the Wiedemann 99 model are detailed in Appendix A. The meaning of parameters CC0-CC9 can be found in the Appendix A. Below are the specific meanings of the four control strategies and practical basis for mapping relationships for real-world vehicle behavior:

Rail Safe: The vehicle travels according to a predetermined specific trajectory and takes stopping measures in case of any conflict. Self-driving cabs can strictly follow the predetermined trajectory of the high-precision map for path planning, and the vehicle will only drive along the predetermined path, avoiding any dynamic adjustments. At intersections or lane-change scenarios, the priority is to stop and wait rather than make active decisions, similar to the “Signal Priority” logic of rail transit. Through high-precision GPS trajectory analysis, the parameters were designed as CC0 = 1.5 m (stationary safety distance) and CC8 = 2 m/s^2^ (conservative acceleration).

Cautious: Accurately calculate time gaps and converge only when they are met. Take deceleration measures to prevent accidents when blind spots are encountered in the detection zone. Self-driving cabs can adopt a conservative following strategy, maintain a safe distance greater than that of a human driver, and actively slow down in blind spots, and extend decision-making time when changing lanes to ensure that surrounding vehicles have sufficient response time. It is parameterized to CC4 = −0.1 m/s (negative velocity difference threshold) in accordance with the blind zone deceleration mechanism.

Normal: Behavioral approaches mimic normal driver behavior, and control can be augmented or diminished depending on how well the detector perceives the surrounding environment. Self-driving cabs can adopt flexible driving strategies that mimic the driving habits of human drivers, allowing for a certain amount of randomness, such as slight trajectory fluctuations when changing lanes. The path is dynamically adjusted within safe limits, such as choosing a detour route in case of congestion or adjusting the following strategy according to the type of vehicle in front. Through human driving simulation and combining the actual following data of Nanjing drivers (average time distance 0.8–1.1 s), the parameter was set to CC1 = 0.9 s (following time distance).

All-Knowing: Ability to accurately sense and predict the surrounding environmental conditions and the behavioral state of other users of the road. Have the ability to apply pressure on other drivers as needed and ensure that no accidents occur. Self-driving cabs can make collaborative decisions, acquire other vehicles’ intentions and traffic light phases through V2X communication to achieve globally optimal path planning, and anticipate potential risks, such as the probability of a vehicle ahead braking sharply, to adjust speed or change lanes in advance. Even in congested scenarios, it can actively compress the distance between the taxi and the vehicle in front of it, prompting the vehicle behind to yield. The global path is optimized for collaborative decision making through dynamic game theory by setting the parameter to CC8 = 4 m/s^2^ (aggressive acceleration).

By controlling these car-following parameters along with other autonomous driving parameters such as penetration rate and number of connected vehicles (robotaxis can communicate with each other to share traffic and driving information to cooperate), we could simulate the traffic operations at specific infrastructures with the presence of robotaxis. While direct validation of AT-specific behaviors remains challenging due to the nascent stage of real-world deployments, our simulation model prioritizes two pillars of credibility: (1) the use of exhaustive, empirically grounded control strategies (via the CoEXist project) to represent AT decision-making, and (2) strict adherence to observed traffic flow patterns and intersection geometries. This approach ensures that the simulated interactions between ATs and human-driven vehicles reflect plausible real-world dynamics, even in the absence of large-scale AT validation data.

### 3.2. Intersection Scenarios

This paper defines two distinct intersection scenarios for analysis. One scenario is modeled after an actual skewed intersection located in Nanjing, China. The other scenario adheres to the Urban Intersection Design Code in China, representing an ideal symmetric intersection.

**Figure 1:**
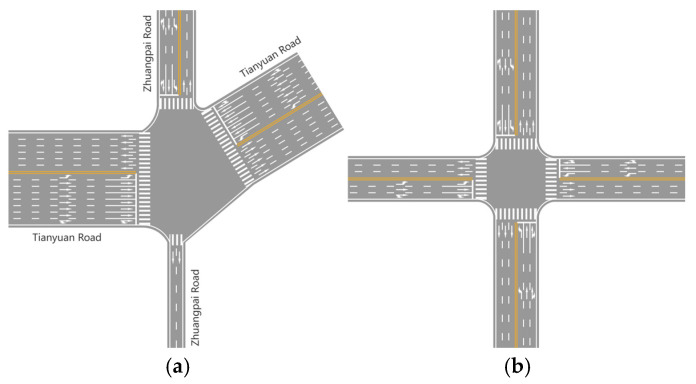
Canalizations of the skewed (**a**) and symmetric (**b**) intersections.

The skewed intersection scenario is based on the actual intersection of Tianyuan Road and Zhuangpai Road in Jiangning District, Nanjing City. Near this intersection, Tianyuan Road acts as a primary thoroughfare, linking the airport expressway to the Dongshan hub and handling a substantial traffic flow. Furthermore, Tianyuan Middle Road is a key route that connects Jiangning with the urban center via Shuanglong Avenue. East of Zhuangpai Road, on the north side of Tianyuan Middle Road, lies a large residential area inhabited by a lot of residents. As a result, a significant influx of private vehicles enters Tianyuan Middle Road and Zhuangpai Road from the north gate of these communities. Situated at the southeast corner of the intersection is the 21st Century International Business Center. The business center’s underground parking entrance and exit, as well as its on-street parking, are located directly across from the intersection. During peak holiday periods, there is a significant influx of motorized and non-motorized vehicles, which considerably affects the traffic flow at the intersection. The intersection is managed by a four-phase traffic signal system, with cycle phases adjusted according to different time periods. The intersection’s layout is depicted in Figure 1.

**Figure 2:**
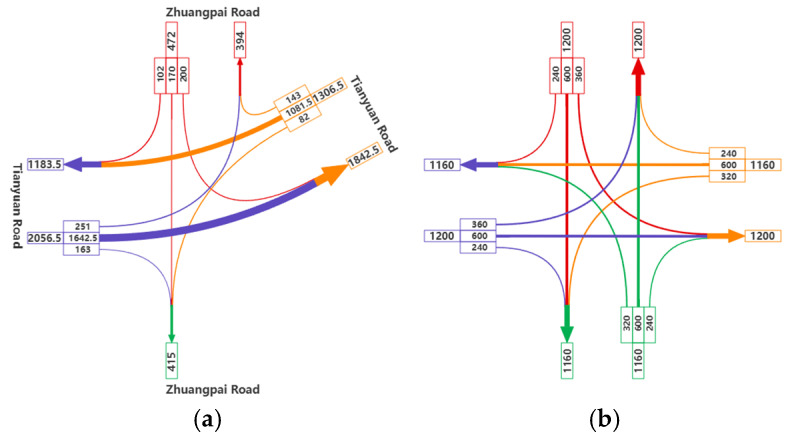
Traffic volume of the skewed (**a**) and symmetric (**b**) intersections.

We have selected the afternoon peak period from 16:15 to 17:15 as the most representative traffic flow at this intersection, based on daily traffic volume. Figure 2 illustrates the spatial distribution pattern of the traffic flow. The signal timing cycle for the intersection of Tianyuan Middle Road and Zhuangpai Road is 177 s, with the following green light durations: 36 s for left-turn traffic on Tianyuan Middle Road, 62 s for through traffic, and 98 s for right-turn traffic; for Zhuangpai Road, the green light duration for through and right-turn traffic is 39 s. Specifically, there are no left turns on Zhuangpai Road.

The symmetric intersection scenario is primarily designed to provide a comparative analysis with the skewed scenario. The symmetric intersection scenario is configured to mirror the skewed one in basic traffic characteristics: It operates with six lanes in each direction. Each approach has three designated lanes: a signal-regulated left-turn lane, a signal-regulated through lane, and a multi-mode third lane. The third lane functions as either a signal-dependent through–right combination lane or an unsignalized dedicated right-turn lane. The major road’s traffic volume is calibrated at 1200 veh/h, incorporating capacity adjustments for the intersection’s skewed geometry. Traffic distribution splits into 30% left turns (phase-controlled), 50% through movements (phase-controlled), and 20% right turns (unsignalized). The signal cycle runs on a fixed 132-s sequence: east–west through movements receive 40 s of green time, north–south through movements also obtain 40 s, while all-direction left turns share a synchronized 20-s green phase. Through traffic benefits from symmetrical signal timing, left turns operate under coordinated four-way phasing, and unchannelized right turns maintain continuous flow via geometric island controls. This configuration balances skewed intersection challenges by dynamically allocating lane functions, prioritizing major road throughput through differential phase durations, and resolving right-turn conflicts via physical channelization.

### 3.3. Safety Effect Evaluation

To evaluate the safety performance of autonomous taxis at skewed intersections, we used the SSAM, a tool developed by the Federal Highway Administration (FHWA). SSAM can identify, classify, and evaluate traffic conflicts within vehicle trajectory data generated by microscopic traffic simulation models, offering safety statistics that assist analysts in designing safer traffic facilities. In this study, the simulation data in VISSIM are exported as trajectory files (*.trj). These files are imported into SSAM to make automatically detections and statistics.

For this study, we have selected two categories of safety statistics to represent safety outcomes: time-based indicators and the number of conflicts. TTC and PET are two widely used time-based indicators for safety measurement. The number of conflicts is categorized into three types: lane-change, rear-end, and path-crossing conflicts. These parameters are detailed in Table 2.

## 4. Results and Discussion

This study focuses on the safety analysis of self-driving cabs at unbalanced intersections, where the core challenges are asymmetric traffic distribution, restricted line-of-sight, and frequent lane-change conflicts. In terms of the relevance of the study metric selection to the study scenario, TTC directly quantifies the urgency of a vehicle approaching a collision, which is particularly applicable to unbalanced intersections with restricted sight lines (e.g., right-turning vehicles suddenly cutting into the straight-ahead flow). However, it is less sensitive to low-speed difference conflicts (e.g., slow lane change) and needs to be combined with other indicators. PET reflects the safety margin after vehicle encroachment and is suitable for evaluating lane change and cross-path conflicts (e.g., left-turning vehicles versus oncoming straight vehicles). The number of conflicts is a macro characterization of the overall conflict frequency, which is suitable for horizontal comparison of the global safety effects of different penetration rates and control strategies.

### 4.1. Time-Based Indicators

**Figure 3:**
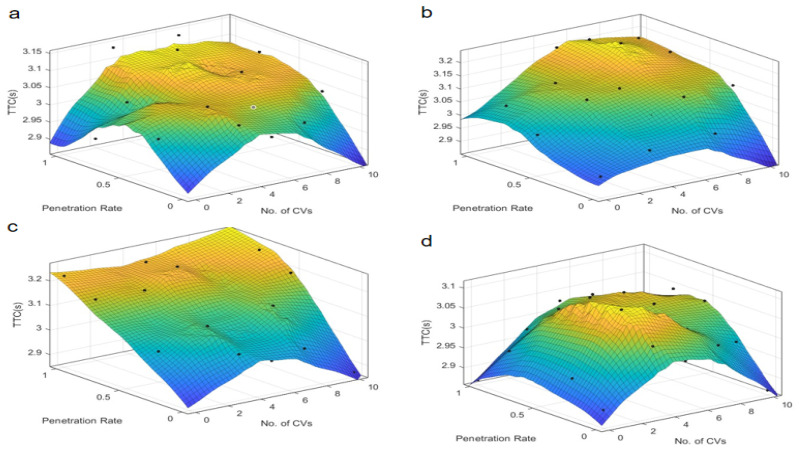
TTC at skewed intersection over penetration rate and number of connected vehicles. Control strategy: (**a**) Rail safe; (**b**) Cautious; (**c**) Normal; (**d**) All-knowing.

**Figure 4:**
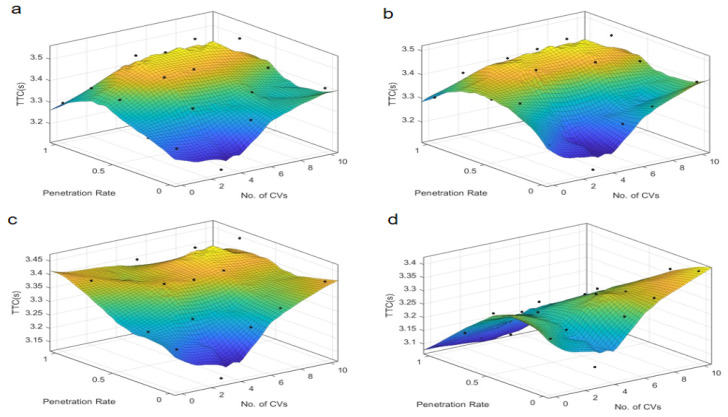
TTC at symmetric intersection over penetration rate and number of connected vehicles. Control strategy: (**a**) Rail safe; (**b**) Cautious; (**c**) Normal; (**d**) All-knowing.

The findings from the time-based safety indicators, TTC and PET, are presented in Figure 3 and Figure 4, which depict the variations of these metrics across different autonomous taxi penetration rates and numbers of connected vehicles at both skewed and symmetric intersections. The results are categorized by autonomous control strategies, ranging from the most conservative “Rail safe” to the most progressive “All-knowing” approaches.

It is observed in Figure 3 that for skewed intersections, an intermediate penetration rate of autonomous taxis appears to yield an optimal safety performance under “Rail safe”, “Cautious”, and “All-knowing” control strategies, indicated by a lower TTC. This is because a moderate number of autonomous taxis can effectively coordinate with each other and with human-driven vehicles, reducing the likelihood of conflicts. However, beyond a certain penetration rate, the complexity of interactions increases, leading to a slight degradation in safety metrics. Conversely, under the “Normal” strategy, TTC increases linearly with the rise in penetration rate, suggesting a potential safety concern with this particular control logic. The linear increase in TTC with the rise in penetration rate under the “Normal” strategy suggests that this control logic, which mimics human driving behavior, may not be as effective in managing the increased complexity of traffic at higher penetration rates. This highlights the need for more advanced control strategies to maintain safety as penetration rates increase. An optimal count of around five connected vehicles is identified, beyond which TTC remains relatively stable. This indicates that a certain level of connectivity is beneficial for safety, but beyond a certain point, additional connectivity does not significantly improve safety metrics. This could be due to the saturation of information exchange and the diminishing returns of increased communication overhead.

Comparatively, Figure 4 illustrates that for symmetric intersections, the TTC is less sensitive to the number of connected vehicles, exhibiting a more proportional relationship with the penetration rate. This suggests that symmetric intersections can better handle increases in penetration rate without significant degradation in safety, likely due to the more uniform traffic distribution and simpler traffic dynamics. Notably, under the “All-knowing” strategy, the TTC is inversely related to the penetration rate, highlighting the complex interplay between automation levels and safety outcomes in traffic management. This is because the “All-knowing” strategy can better predict and manage traffic interactions, reducing the likelihood of conflicts even as the number of autonomous taxis increases.

**Figure 5:**
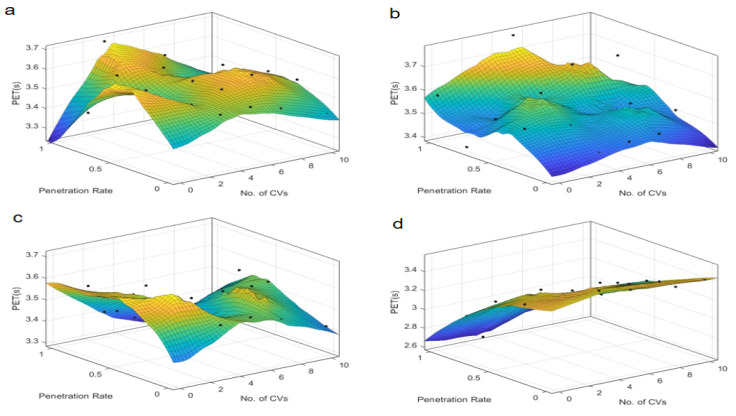
PET at skewed intersection over penetration rate and number of connected vehicles. Control strategy: (**a**) Rail safe; (**b**) Cautious; (**c**) Normal; (**d**) All-knowing.

**Figure 6:**
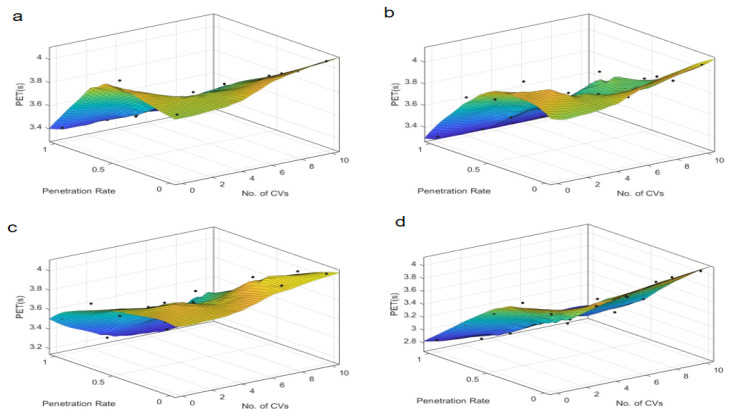
PET at symmetric intersection over penetration rate and number of connected vehicles. Control strategy: (**a**) Rail safe; (**b**) Cautious; (**c**) Normal; (**d**) All-knowing.

Further analysis of the Post-Encroachment Time (PET), as depicted in Figure 5 and Figure 6, reveals distinct patterns compared to TTC. At skewed intersections, the PET indicates an optimal penetration rate near 0.4 under “Rail safe”, “Cautious”, and “Normal” control strategies, where safety is maximized. This suggests that a moderate penetration rate allows for better coordination and reaction times, maximizing safety margins. However, the “All-knowing” strategy exhibits an inverse relationship with PET, indicating that higher penetration rates could be detrimental to safety under this control logic. This could be due to the increased complexity of interactions and the potential for over-reliance on advanced decision-making algorithms, which may not always account for all possible scenarios. In contrast, the “All-knowing” strategy exhibits an inverse relationship with PET, suggesting that higher penetration rates could be detrimental to safety under this control logic. For symmetric intersections, the PET is inversely proportional to the penetration rate, indicating that as the number of autonomous taxis increases, the safety margin, as measured by PET, decreases. This suggests that while symmetric intersections can handle higher penetration rates, the increased number of autonomous taxis can still lead to a reduction in safety margins, highlighting the need for careful management of penetration rates and control strategies. This inverse relationship underscores the need for a nuanced approach to autonomous vehicle deployment, where the benefits of increased penetration are weighed against potential safety risks.

The number of connected vehicles also plays a pivotal role in safety outcomes. At symmetric intersections, connectivity seems to have a less pronounced effect on PET, suggesting that the inherent safety benefits of autonomous vehicles may be more robust to variations in connectivity. However, at skewed intersections, an optimal number of connected vehicles is identified around 5 under “Rail safe”, “Cautious”, and “Normal” strategies, beyond which the PET remains relatively neutral, indicating a saturation point in the safety benefits of increased connectivity. This suggests that while connectivity is beneficial, there is a saturation point beyond which additional connectivity does not significantly improve safety margins. Under the “All-knowing” strategy, the impact of connectivity on PET is minimal, which may be attributed to the advanced decision-making capabilities of the control strategy that can compensate for variations in connectivity.

These findings suggest that the deployment of autonomous taxis should consider the specific characteristics of the intersection, such as skewed intersections, that may require a more tailored approach to maximize safety. The results also highlight the importance of selecting an appropriate control strategy that aligns with the intersection’s traffic dynamics and the level of vehicle connectivity. “Rail safe” prioritizes collision avoidance through strict adherence to safety distances (CC0 = 1.5 m) and low acceleration (CC8 = 2 m/s^2^). At low penetration rates (0.3–0.5), conflicts are reduced by enforcing predictable AT behavior. At high penetration (>0.7), overcautious autopilot cabs create an “accordion effect” (stop-and-go) that increases rear-end conflicts. So, safety metrics (TTC, PET) are negatively correlated with travel density due to the conservative logic that exacerbates congestion. It is prioritizing safety but exacerbating congestion at high penetration rates, which offsets safety gains. Differently “All-knowing” performs perfect V2X coordination by actively optimizing the driving trajectory (CC0 = 1 m, CC8 = 4 m/s^2^). At low penetration rates (0.3–0.5), the limited scope of coordination leads to sub-optimal safety gains. At high penetrations (>0.7), system-wide coordination minimizes conflicts, but there is a risk of over reliance on connectivity. Safety metrics (TTC, PET) are positively correlated with travel density Because high penetration allows for system-wide optimization. It utilizes system-wide coordination but requires critical penetration thresholds. Therefore, there is a need for an adaptive deployment framework. The interplay between penetration rate, connectivity, and control strategy is complex and requires careful consideration to ensure that the integration of autonomous taxis enhances safety without compromising efficiency.

### 4.2. Number of Conflicts

**Figure 7:**
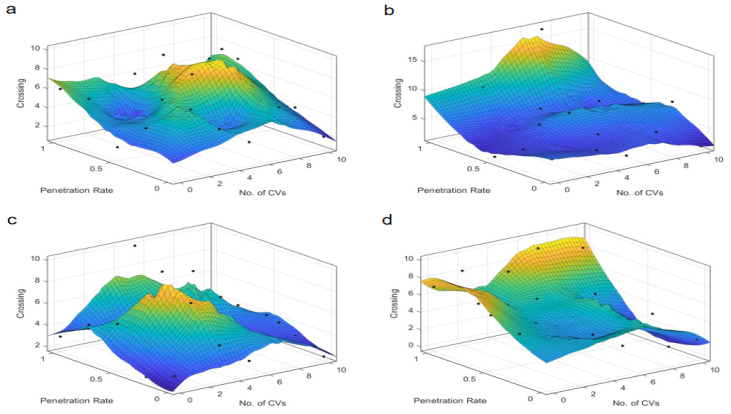
Crossing conflicts at skewed intersection over penetration rate and number of connected vehicles. Control strategy: (**a**) Rail safe; (**b**) Cautious; (**c**) Normal; (**d**) All-knowing.

**Figure 8:**
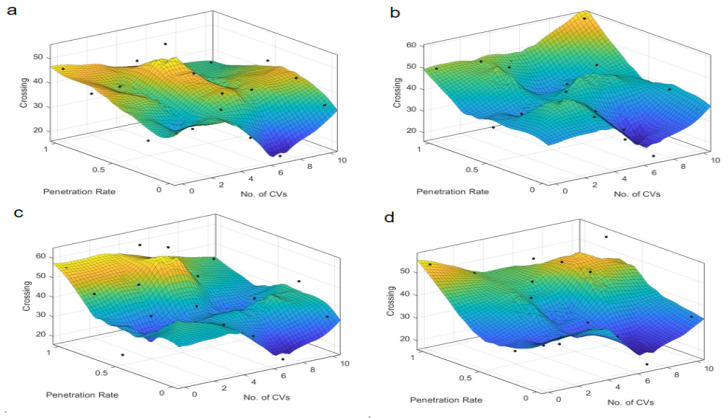
Crossing conflicts at symmetric intersection over penetration rate and number of connected vehicles. Control strategy: (**a**) Rail safe; (**b**) Cautious; (**c**) Normal; (**d**) All-knowing.

**Figure 9:**
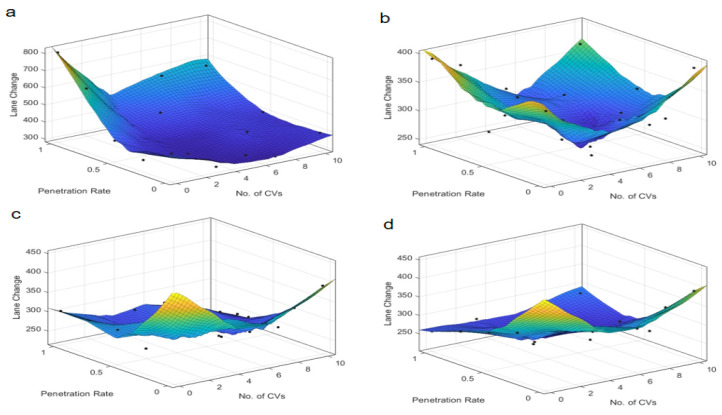
Lane-change conflicts at skewed intersection over penetration rate and number of connected vehicles. Control strategy: (**a**) Rail safe; (**b**) Cautious; (**c**) Normal; (**d**) All-knowing.

**Figure 10:**
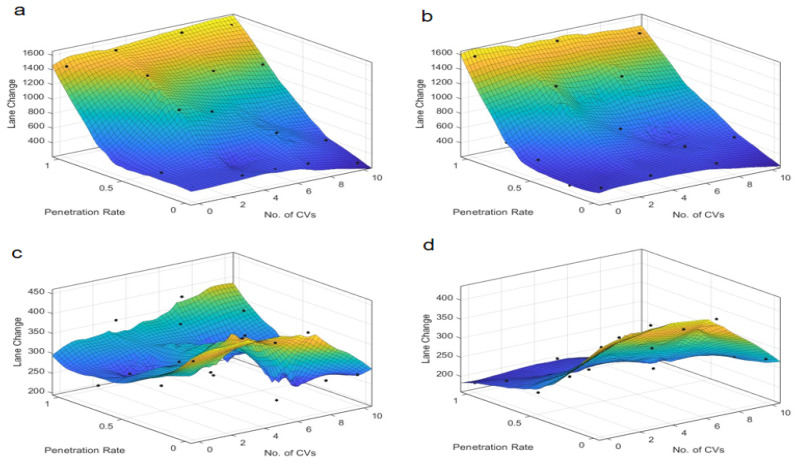
Lane-change conflicts at symmetric intersection over penetration rate and number of connected vehicles. Control strategy: (**a**) Rail safe; (**b**) Cautious; (**c**) Normal; (**d**) All-knowing.

**Figure 11:**
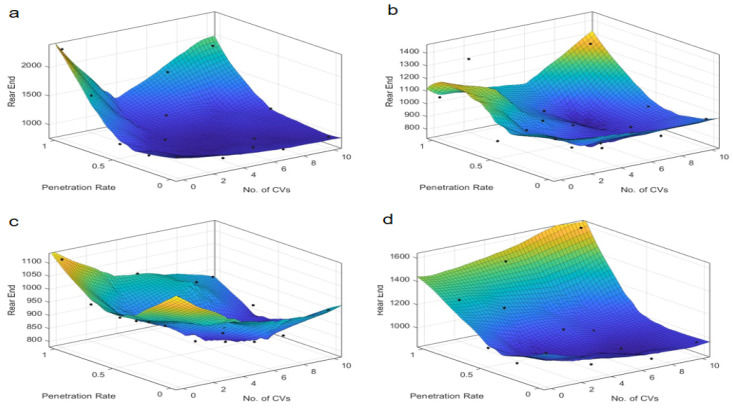
Rear-end conflicts at skewed intersection over penetration rate and number of connected vehicles. Control strategy: (**a**) Rail safe; (**b**) Cautious; (**c**) Normal; (**d**) All-knowing.

**Figure 12:**
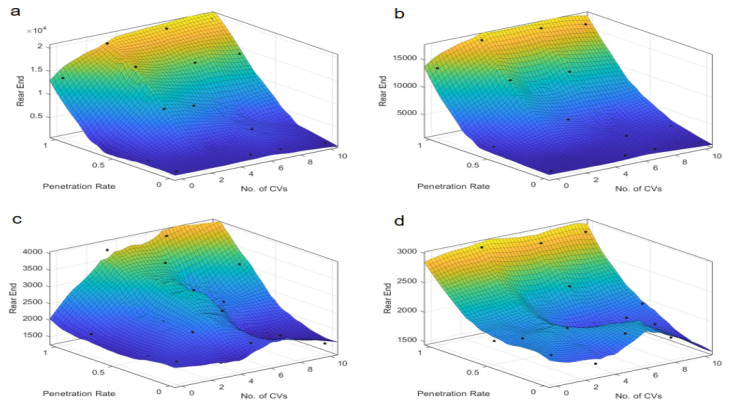
Rear-end conflicts at symmetric intersection over penetration rate and number of connected vehicles. Control strategy: (**a**) Rail safe; (**b**) Cautious; (**c**) Normal; (**d**)All-knowing.

The examination of conflict types, specifically crossing, lane-change, and rear-end conflicts, provides a granular understanding of the safety implications at intersections, as illustrated in Figure 7, Figure 8, Figure 9, Figure 10, Figure 11 and Figure 12. For crossing conflicts, the data reveal a convex relationship at skewed intersections. The convex relationship suggests that an intermediate penetration rate initially reduces safety due to the complexity of mixed traffic dynamics, but safety improves as the penetration rate increases further. This is likely because a higher penetration rate allows for better coordination and communication among autonomous taxis, reducing the likelihood of crossing conflicts. Conversely, symmetric intersections display a concave pattern, with an optimal safety zone around a 0.4–0.6 penetration rate. The concave pattern indicates that symmetric intersections can maintain a higher level of safety within a specific penetration rate range, but safety may degrade outside this range. This suggests that symmetric intersections are more robust to changes in penetration rate, but there is still an optimal range for safety. This divergence underscores the unique challenges posed by skewed intersections and the potential for tailored control strategies to mitigate safety risks. At skewed intersections, the lack of signal control in the dedicated right-turn lane forces merging vehicles to compete with straight-line vehicles. Symmetrical intersections, on the other hand, have an even distribution of conflicts due to balanced sight lines and right-turn signal control.

Lane-change conflicts exhibit a dichotomy based on the radicalness of the control strategy. Less radical strategies, such as “Rail safe” and “Cautious”, correlate with an increase in conflict numbers as penetration rates rise, indicating a potential safety concern with higher levels of autonomy. This is because less radical strategies may not effectively manage the increased complexity of lane-change maneuvers in mixed traffic, leading to potential safety concerns. In stark contrast, more radical strategies like “Normal” and “All-knowing” demonstrate a decrease in lane-change conflicts with increased penetration. This suggests that advanced control logic can better manage lane-change dynamics, reducing the likelihood of conflicts and enhancing safety. Due to the uneven lane distribution at skewed intersections, alternating through right lanes at skewed intersections forces frequent lane changes for right-turning operations. At the same time, the phase mismatch causes the signal phasing to prioritize straight through traffic, compressing the lane change window for turning vehicles. The symmetrical layout reduces the need to change lanes by dedicating turn lanes to the turning traffic.

Rear-end conflicts at skewed intersections present an intriguing case, an optimal penetration rate is identified, which is inversely proportional to the number of conflicts. This suggests that a certain threshold of autonomous vehicle presence can significantly reduce rear-end collision risks by improving coordination and reaction times. At symmetric intersections, the almost linear relationship between penetration rate and rear-end conflicts indicates that the integration of autonomous taxis can universally reduce rear-end collision risks in symmetric traffic conditions. This is likely due to the more uniform traffic distribution and simpler traffic dynamics, which allow for better management of following distances and speeds. Longer approach lanes and speed bumps at skewed intersections create sudden speed differentials. In terms of signal coordination: Longer green phases on arterials result in queuing for traffic, increasing the risk of rear-end collisions during deceleration.

The results indicate that the optimal penetration rate for safety at skewed intersections is around 0.5–0.7 for most control strategies. The identified penetration rate range reflects an empirical “safety-optimal” zone where conflict metrics show consistent improvement compared to lower or higher rates. While this range is derived from observed trends rather than formal optimization, it highlights a critical threshold for balancing safety and efficiency in AT deployment. This is because a moderate-to-high penetration rate allows for better coordination and communication among autonomous taxis, reducing the likelihood of conflicts. However, beyond a certain point, the increased complexity of interactions can lead to a slight degradation in safety metrics. For symmetric intersections, the trend suggests that higher penetration rates and connectivity levels may lead to an increase in conflicts, which could be mitigated by refining control strategies. The choice of control strategy significantly influences safety outcomes. Less radical strategies may not be as effective in managing complex traffic dynamics, while more radical strategies can enhance safety by better managing lane-change and rear-end conflict scenarios. The skewed intersection’s geometry—unsignalized right-turn lanes, asymmetric sightlines, and elongated deceleration zones—directly amplifies crossing and rear-end conflicts. Lane-change conflicts, meanwhile, are driven by phase mismatches and irregular lane allocations. The “All-knowing” strategy, in particular, shows significant benefits in reducing TTC and PET, likely due to its advanced decision-making capabilities. Skewed intersections present unique challenges and require tailored control strategies to mitigate safety risks. Symmetric intersections, on the other hand, can benefit from higher penetration rates and connectivity, but the deployment strategy should still consider the specific traffic dynamics to maximize safety and efficiency. These insights are crucial for the development of traffic management policies that prioritize safety and efficiency, particularly in the context of skewed intersections where the interplay between autonomous vehicle deployment and traffic dynamics is more pronounced.

While our study prioritizes the exploration of AT deployment strategies under calibrated parameters, future work could expand the scope of sensitivity analyses to include additional variables such as heterogeneous driver behaviors and communication latency. However, the qualitative stability of our findings—evidenced by consistent safety trends across control strategies and intersection types—supports the practical relevance of our conclusions for skewed intersection management.

## 5. Conclusions

This study optimizes autonomous taxi deployment strategies for safety at skewed intersections through simulation-based analysis, revealing the complex interplay between penetration rates, control strategies, and geometric characteristics. Our findings demonstrate that skewed intersections achieve optimal safety performance (as measured by TTC and PET) at an intermediate AT penetration rate of 0.5–0.7. Exceeding this range risks degraded safety due to coordination complexity or overreliance on connectivity. Control strategies critically influence outcomes: proactive approaches like “All-knowing” enhance conflict resolution through predictive coordination, whereas conservative strategies, such as “Rail-safe”, may inadvertently exacerbate congestion at high penetration rates. Furthermore, the asymmetric geometry of skewed intersections—manifested in unsignalized right-turn conflicts—demands tailored deployment strategies, while symmetric intersections exhibit more linear safety–penetration relationships.

To translate these findings into real-world applications, a synergistic framework integrating technological advancements and policy adaptation is essential. Technologically, deploying 5G-V2X communication infrastructure and edge computing platforms can enable real-time environmental awareness and cooperative decision-making required for strategies like “All-knowing”. However, challenges such as communication latency (>100 ms) and sensor occlusion necessitate redundancy protocols (e.g., fallback to “Cautious” mode in low-connectivity scenarios). Infrastructure upgrades, including dynamic priority lanes and V2X-enabled traffic signals synchronized with intersection geometries, could enforce optimal penetration rates during peak hours. Policy-wise, revising traffic codes to mandate AT-specific safety margins (e.g., conflict resolution protocols) and establishing certification standards for decision-making algorithms, validated through SSAM-based conflict analysis, are imperative. Pilot programs at high-density skewed intersections, such as Nanjing’s Tianyuan–Zhuangpai intersection, could iteratively validate safety improvements through phased AT deployment while mitigating public acceptance risks.

This study assumes idealized connectivity and homogeneous AT behaviors, whereas real-world heterogeneity, including sensor noise, mixed road users, and adversarial scenarios (e.g., sudden pedestrian intrusions), may alter conflict dynamics. Future work should prioritize robustness testing against such conditions and develop adaptive fleet management algorithms for dynamic penetration rate control. Bridging the gap between simulation and practice will require continuous collaboration among policymakers, technologists, and urban planners to ensure the safe and efficient integration of autonomous mobility systems.

## Figures and Tables

**Table 1:** Car-following Parameters Defined by CoEXist [27].

Parameter	Rail Safe	Cautious	Normal	All-Knowing	HV
CC0	1.5	1.5	1.5	1	1.5
CC1	1.5	1.5	0.9	0.6	0.9
CC2	0	0	0	0	4
CC3	−10	−10	−8	−6	−8
CC4	−0.1	−0.1	−0.1	−0.1	−0.35
CC5	0.1	0.1	0.1	0.1	0.35
CC6	0	0	0	0	11.44
CC7	0.1	0.1	0.1	0.1	0.25
CC8	2	3	3.5	4	3.50
CC9	1.2	1.2	1.5	2	1.50
Maximum deceleration	−4/−3	−3.5/−2.5	−4/−3	−4/−4	−4/−3
−1 m/s per distance	100/100	80/80	100/100	100/100	100/100
Accepted deceleration	−1/−1	−1/−1	−1/−1	−1/−1.5	−1/−1
Min. headway (front/rear)	1	0.5	0.5	0.5	0.5
Max. deceleration for cooperative braking	−2.5	−3	−6	−3	−3
Behavior at amber signal	continuous check	continuous check	one decision	one decision	continuous check
Reduced safety distance factor	1	1	1	1	0.6
Reduced safety start upstream of stop line	100	100	100	100	100
Reduced safety end upstream of stop line	100	100	100	100	100

**Table 2:** Safety-effect-evaluation parameters.

	Parameter Name	Description	Units
Time-based Indicators	TTC	Time-to-Collision: The minimum time before a potential collision occurs.	Seconds
	PET	Post-Encroachment Time: The minimum time after an encroachment on the traffic space before a potential collision.	Seconds
Number of Conflicts	NoLC	Number of Lane-Change Conflicts: The count of conflicts arising from lane-changing maneuvers.	Counts
	NoRE	Number of Rear-End Conflicts: The count of conflicts involving potential rear-end collisions.	Counts
	NoPC	Number of Path-Crossing Conflicts: The count of conflicts involving path crossings.	Counts

## Data Availability

The data in this work is available upon request.

## Appendix A

**Table A1:** Meaning of car-following parameters in Wiedemann 99 model [28].

Parameters	Unit	Description
CC0	m	Standstill distance: The desired standstill distance between two vehicles. No stochastic variation.
CC1	s	Gap time distribution: Time distribution from which the gap time in seconds is drawn which a driver wants to maintain in addition to the standstill distance.
CC2	m	“Following” distance oscillation: Maximum additional distance beyond the desired safety distance accepted by a driver following another vehicle before intentionally moving closer.
CC3	s	Threshold for entering “BrakeBX”: Time in seconds before reaching the maximum safety distance (assuming constant speed) to a leading slower vehicle at the beginning of the deceleration process (negative value).
CC4	m/s	Negative speed difference: Lower threshold for relative speed compared to slower leading vehicle during the following process (negative value).
CC5	m/s	Positive speed difference: Relative speed limit compared to faster leading vehicle during the following process (positive value).
CC6	1/(m·s)	Distance impact on oscillation: Impact of distance on limits of relative speed during following process: Value 0: Distance has no impact on limits. Larger values: Limits increase with increasing distance.
CC7	m/s^2^	Oscillation acceleration: Acceleration oscillation during the following process.
CC8	m/s^2^	Acceleration from standstill: Acceleration when starting from standstill. Is limited by the desired and maximum acceleration functions assigned to the vehicle type.
CC9	m/s^2^	Acceleration at 80 km/h: Acceleration at 80 km/h is limited by the desired and maximum acceleration functions assigned to the vehicle type.
